# Prevention of Premature Ovulation by Administration of Gonadotropin Releasing Hormone Antagonist the day After Ovulation Triggering in Diminished Ovarian Reserve Patients

**DOI:** 10.1055/s-0041-1736297

**Published:** 2022-02-09

**Authors:** Bulat Aytek Sik, Ozan Ozolcay, Yilda Arzu Aba, Alper Sismanoglu, Sifa Savas, Serkan Oral

**Affiliations:** 1 Department of Reproductive Endocrinology and Infertility, Sisli Kolan International Hospital, Istanbul, Turkey; 2 Department of Reproductive Endocrinology and Infertility, Istanbul IVF Centre, Istanbul, Turkey; 3 Faculty of Health Sciences, Bandirma Onyedi Eylül University, Balıkesir, Turkey

**Keywords:** diminished ovarian reserve, GnRH antagonist, M _2_ oocyte, premature ovulation, pregnancy rates, reserva ovariana diminuída, antagonista de GnRH, oócito M2, ovulação prematura, taxas de gravidez

## Abstract

**Objective** The aim of the present retrospective study was to investigate the effectiveness of single-dose gonadotropin releasing hormone (GnRH) antagonist administration, the day after human chorionic gonadotropin (hCG) triggering for final oocyte maturation, on the prevention of premature luteinization in patients with diminished ovarian reserve in in-vitro fertilization (IVF) cycles. The secondary objective of the study was to search the effect of this protocol on pregnancy outcomes.

**Methods** This is a retrospective study including 267 infertile patients who have single antral follicle seen with ultrasonography on the 2 ^nd^ or 3 ^rd^ day of the menstrual cycle before starting IVF treatment. We randomized patients into two groups. The case group comprised patients who had single-dose GnRH antagonist injection the day after hCG triggering formed, and the patients who had the standard treatment regime formed the control group. In both groups, the oocytes were collected 36 hours after hCG injection.

**Results** The premature ovulation rate was significantly low in the case group compared with the control group (6.86 versus 20.6% per scheduled cycle) ( *p* = 0.022). Also, the oocyte retrieval rate (93.14 versus 67.87% per scheduled cycle) ( *p* = 0.013), the oocyte maturity rate (79.42 *versus* 47.87%) ( *p* = 0.041), the fertilization rate (65.68 *versus* 34.54%) ( *p* = 0.018), and the embryo transfer rate per scheduled cycle (44.11 versus 18.78%) ( *p* = 0.003) were higher in the GnRH antagonist group than in the control group.

**Conclusion** The administration of GnRH antagonist the day after hCG trigger in IVF treatments of patients with diminished ovarian reserve enabled a significant decrease in the rate of premature ovulation but had no effect on live birth rate.

## Resumo

**Objetivo** O objetivo do presente estudo retrospectivo foi investigar a eficácia da administração do antagonista do hormônio liberador da gonadotrofina (GnRH) em dose única no dia seguinte ao desencadeamento da gonadotrofina coriônica humana (hCG) para a maturação final do oócito, na prevenção da luteinização prematura em pacientes com diminuição do ovário reserva em ciclos de fertilização in vitro (FIV). O objetivo secundário do estudo foi pesquisar o efeito deste protocolo nos resultados da gravidez.

**Métodos** Trata-se de um estudo retrospectivo incluindo 267 pacientes inférteis que apresentam um único folículo antral visto por ultrassonografia no 2° ou 3° dia do ciclo menstrual antes de iniciar o tratamento de FIV. Nós randomizamos os pacientes em dois grupos. Os pacientes que receberam injeção de antagonista de GnRH em dose única no dia seguinte ao desencadeamento do hCG formaram o grupo caso, e os pacientes que receberam o regime de tratamento padrão formaram o grupo controle. Em ambos os grupos, os oócitos foram coletados 36 horas após a injeção de hCG.

**Resultados** A taxa de ovulação prematura foi significativamente baixa no grupo caso em comparação com o grupo controle (6,86 *versus* 20,6% por ciclo programado) ( *p* = 0,022). Além disso, a taxa de recuperação de oócitos (93,14 *versus* 67,87% por ciclo programado) ( *p* = 0,013), a taxa de maturidade do oócito (79,42 *versus* 47,87%) ( *p* = 0,041), a taxa de fertilização (65,68 *versus* 34,54%) ( *p* = 0,018) e a taxa de transferência de embriões por ciclo programado (44,11 *versus* 18,78%) ( *p* = 0,003) foram maiores no grupo antagonista de GnRH do que no grupo controle.

**Conclusão** A administração de antagonista de GnRH, no dia seguinte ao desencadeamento de hCG em tratamentos de FIV de pacientes com reserva ovariana diminuída permitiu uma redução significativa na taxa de ovulação precoce, mas não teve efeito na taxa de nascidos vivos.

## Introduction

Early decrease is detected in the ovarian reserve in a mean of 10% of women due to various reasons regardless of age. Diminished ovarian reserve is determined in women with low antral follicle count and, therefore, with low response to ovarian stimulation and low fecundity compared with other women of their age despite having regular menstrual cycles. 1 The increase of premature luteinization in patients with diminished ovarian reserve (particularly with a single oocyte) causes either cycle cancellation or early ovulation in the oocyte pick-up (OPU) period. 2 Gonadotropin-releasing hormone (GnRH) antagonists have recently been administered to avoid premature luteinizing hormone (LH) increase, and luteinization in the controlled ovarian hyperstimulation (COH) performed in in vitro fertilization (IVF) treatments. 3 Gonadotropin releasing hormone (GnRH) antagonists, particularly in comparison with the GnRH agonist protocols, decrease the LH fluctuations more safely and effectively, in a shorter period, with fewer injections, and with less adverse effects. 4 Flare-up effect doesn't occur due to hypo estrogenic side effect, shorten the cycle period, decrease the amount of gonadotrophine dosage, immediate effect and directly binding to GnRH receptors in the pituitary gland were the most significant advantage of GnRH antagonist protocol. Also the suppression ofLH secretion happens in a short period of time. 4 5 Although these advantages are also important for patients with normal ovarian reserve, they are more important in patients with diminished ovarian reserve. 6

The aim of the present retrospective study was to analyze the effectiveness of single dose of GnRH antagonist injection 24 hours after hCG trigger in 267 infertile patients with only one antral follicle. The primary objective was to observe if this protocol decreases the premature ovulation, and the secondary goal was to search its effects on pregnancy results.

## Methods

This is a retrospective study including 267 infertile patients aged between 25 and 35 years old who were referred to the IVF Unit of the Istanbul IVF Center with the problem of infertility related to diminished ovarian reserve between January 2016 and May 2017, and who were detected as having a single antral follicle during the transvaginal ultrasonography (TVUSG) evaluation on the 2 ^nd^ or 3 ^rd^ day of their menstrual cycle. All the patients refused oocyte donation. Institutional Review Board approval was not required for the study due to its retrospective nature and to the fact that study data was constantly managed in a way that excluded the identification of subjects. Informed consent was obtained from all patients. Patients with male factor infertility, patients with > 1 antral follicle, and patients > 35 years old were excluded from the study. Fresh embryo transfer was performed to all the patients in the study.

Folliculometric measurement was performed with TVUSG on the 2 ^nd^ or 3 ^rd^ day of the menstrual cycle of 267 patients in the case and control groups. The treatment was initiated with 2.5 mg oral letrozole administration (Femara tablet, Novartis, Italy) twice daily for 5 days. Controlled ovarian hyperstimulation was initiated with human menopausal gonadotropin (hMG) injection 150 IU/day (Menopur 75 IU SC/IM, Ferring, USA). Cetrorelix 0.25 mg injection (GnRH antagonist) (Cetrotide 0.25 mg, Merck, Greece) was added to the regime when the follicle diameter was ≥ 12 mm. The patients were monitored with TVUSG on alternate days until the follicle diameter reached between 18 and 19 mm. When the follicle diameter was between 18 and 19 mm, ovulation triggering was done with 5,000 units of IM/SC hCG (Choriomon 5000 IU, IBSA, Italy) injection and oocyte pickup was arranged 36 hours after hCG injection. A total of 102 patients, who were selected using the systematic randomization method, were administered a single dose of GnRH antagonist the day after hCG administration, and these patients constituted the case group. The control group included 165 patients who were administered the standard treatment regime with hCG trigger. The oocyte pickup procedure was performed by a single physician using a 17-G fine oocyte aspiration needle (Cook Medical, Brisbane, Australia) with TVUSG guidance and under sedative anesthesia. The egg retrieval time was 36 hours after hCG administration for the patients in both groups.

The obtained oocytes were separated with the denudation procedure using hyalarunidase (Life Global, Canada) from their surrounding cumulus 2 hours after collection. Intracytoplasmic sperm injection (ICSI) was performed to mature oocytes (metaphase II) after denudation. Normally fertilized zygotes, which were observed as 2PN (pronucleus) appearance 24 hours after ICSI, were cultured for 3 days in the culture medium (Life Global, Canada). All embryos were cultured in multigas incubators (Hessen, Almanya) at 37°C, which provided the environment of 6% CO _2_ and 5% O _2._ On day 3, fresh embryo transfer was performed to all the patients in the study. Embryo transfer was completed with the exact placement of the embryo transvaginally to the mid-uterus by a single physician under TVUSG guidance. Luteal phase support was initiated in all patients after embryo transfer.

**Fig. 1:**
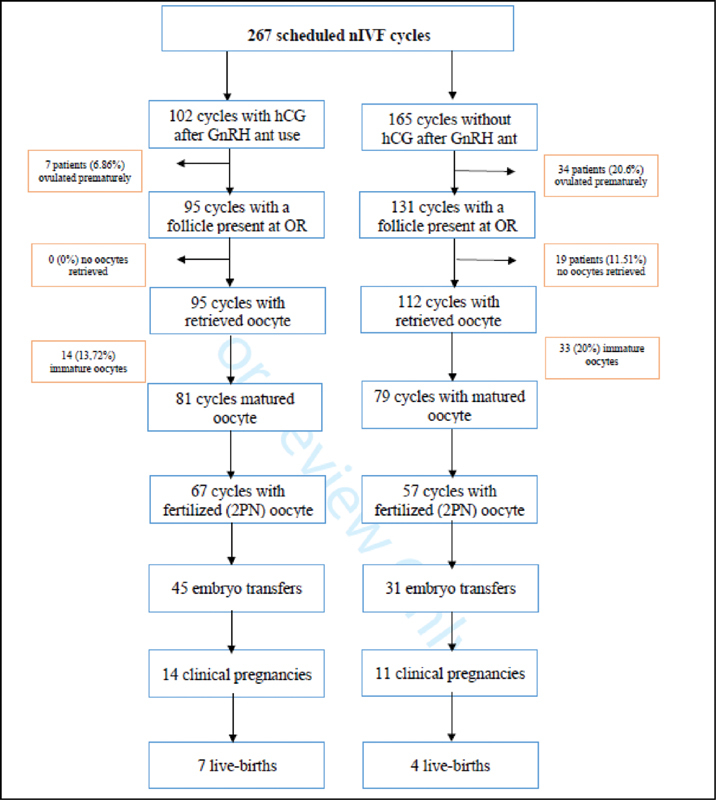
Consort flow diagram.

The rates of premature ovulation and fresh embryo transfer are the indicators evaluated in the first phase for each cycle. The indicators evaluated in the second phase are clinical pregnancy rate and live birth rate per embryo transfer. Clinical pregnancy was described as positive with the detection of a gestational sac on the 6 ^th^ week of pregnancy using TVUSG. Pregnancy loss before the 22 ^nd^ week of pregnancy was described as missed abortion. The treatment process of the patients is shown in the flow diagram in Fig. 1 .

### Statistical Analysis

SPSS for Windows version 11.0 (SPSS Inc., Chicago, IL, USA) was used for the statistical analysis of the present study. The Student *t* -test, the Mann-Whitney U test, the paired *t* -test, the Wilcoxon rank test, the Fisher exact test, and the chi-squared test were used for comparisons. Statistical significance was set at *p* < 0.05. The association between GnRH antagonist administration and premature ovulation rate and the rate of embryo transfer per scheduled cycle was evaluated (adjusted for patient age, oestradiol concentration and follicular size on triggering day, and time interval between triggering and oocyte retrieval time).

## Results

The results of 102 patients (38.2%) in the study group who were administered single-dose GnRH antagonist the day after hCG administration, and those of 165 patients (61.8%) who were administered standard treatment were compared; the results of the IVF cycles are summarized in Table 1 .

**Table 1:** Comparison of the main characteristics and clinical results of the study and control groups

Study Groups	GnRH after hCG Group	Standard therapy group	*p-value*
Scheduled cycles	102 (38.2%)	163 (61.8%)	−
Patient age (range 30–35 years old)	32.9 ± 1.8	33.6 ± 0.9	0.7821*
On triggering day			
LH (IU/mL) (range 10–30)	4,6 ± 0.7	4,9 ± 0.9	0.510
Estradiol (IU/mL) (hCG day)	178 ± 58.2	174 ± 67.3	0.608*
Time between triggering and OR (h)	36.0 ± 0.5	36.0 ± 0.5	0.998*
Premature ovulation a	7 (6.86%)	34 (20.6%)	0.022**
Retrieved oocytes a	95 (93.14%)	112 (67.87%)	0.013**
Mature oocytes a	81 (79,42%)	79 (47.87%)	0.041**
Fertilized (2PN) oocytes a	67 (65,68%)	57 (34.54%)	0.018**
Embryo transfers a, b	45 (44,11%)	31 (18.78%)	0.003**
Clinical pregnancies c	14 (13,72%)	11(6.66%)	0.058**
Live birth a	7 (6.86%)	4 (2.42%)	0.062**

Abbreviations: hCG, human chorionic gonadotropin; LH, luteinizing hormone; OR, oocyte retrieval.

a Percentage of scheduled cycles.

b Summing fresh and vitrified/warmed embryo transfers.

c Percentage of embryo transfers.

* Independent *t* -test.

** Chi-squared test.

A statistically significant decrease in the premature ovulation rate (6.86 versus 20.6% per scheduled cycle) ( *p* = 0.022) and a significant increase in the oocyte retrieval rate (93.14 versus 67.87% per scheduled cycle) ( *p* = 0.013) were observed in the case group compared with the control group. In addition, a higher number of mature oocytes (79.42 versus 47.87%) ( *p* = 0.041) and fertilized (2PN) oocytes (65.68 versus 34.54%) ( *p* = 0.018) were obtained in the case group. A higher number of embryo transfers could be performed in the case group (44.11 versus 18.78%) ( *p* = 0.003). However, no significant difference was detected between the case and control groups regarding the rates of clinical pregnancies and live births ( *p* > 0.05).

There were no statistically significant differences between the case and control groups regarding estradiol levels (178 ± 58.2 IU/ml versus 174 ± 67.3 IU/ml) ( *p* = 0.6082) and LH levels (4.6 ± 0.7 IU/ml versus 4,9 ± 0.9 IU/ml) ( *p* = 0.510) measured on the day of ovulation triggering. In addition, no difference was observed in gestational age at delivery (37.6 ± 1.5 versus 37.9 ± 1.3 weeks) and birthweight of singleton newborns (2,786 ± 417 versus 2,893 ± 510 grams) between both groups.

## Discussion

During IVF treatments with GnRH antagonist protocols, all of us sometimes observe premature increase in LH levels before ovulation triggering. This LH increase ends with cycle cancellation or premature ovulation, both of which are very frustrating for the patients as well as for the clinicians. There is a higher cancellation rate because of premature LH increase in diminished ovarian reserve patients compared with patients with normal ovarian reserve. The risk factors for this premature luteinization in diminished ovarian reserve patients are not still clearly defined. 7 8 The aim of GnRH antagonist administration during the controlled ovarian stimulation cycles is to decrease this LH surge before the ovulation triggering. Luteinizing hormone is essential for the follicular growth and final maturation and ovulation of the oocyte. 9 Luteinizing hormone causes the increase in the androgen secretion from theca cells and, thus, increases the estradiol concentration of the follicle. 10 Luteinizing hormone in late follicular phase helps the production of small amounts of progesterone, which also triggers follicular growth and maturation enhancing positive estrogen feedback. 11 Many studies showed the importance of LH levels on follicular growth and concomitant clinical results during controlled ovarian stimulation. 12 13 Fluctuations of LH levels during late follicular phase have an important role on functional and morphologic changes of the oocytes. It also influences the meiotic and fertilization potential of the oocytes. 14 The GnRH antagonist protocols have been used commonly in the last years for IVF treatments. 15 This agent binds to GnRH receptors in the pituitary gland, thus effectively inhibiting LH secretion, and this is how they block premature luteinization and ovulation in IVF cycles. 16

In addition to a significant decrease in early premature ovulation in the case group for whom we aimed to decrease the premature ovulation risk, a significant increase was also detected in the number of the collected oocytes, mature oocytes, and in the fertilized (2PN) oocyte rates. The detected embryo transfer rate per scheduled cycle was also higher in the case group. However, these results caused no statistically significant difference regarding the rates of clinical pregnancies and live births.

Patients with diminished ovarian reserve represent the group in whom successful treatment is difficult to accomplish during assisted reproduction techniques due to the limited number of ova and embryos, and with low rates of pregnancy. 17 Also, it was demonstrated in large scaled studies that the rates of pregnancies and live births were lower in patients from whom ≤ 3 oocytes were obtained, independent of the treatment protocol and age group. 18 Similarly, it was found in another study that there was no difference in the rates of live births in different groups aged > and < 40 years old, and the only factor correlated with the rate of live births was the number of oocytes. 19 We also aimed to obtain more successful cycles in these patient groups, and to be able to increase the number of collected oocytes.

The major problem that may be encountered in patients who are planned to undergo IVF in a natural cycle is early ovulation. The efficacy of natural-cycle IVF is inhibited due to high rates of cancellations because of premature LH increase and premature ovulation. Pelinck et al. 2 emphasized that it was difficult to control spontaneous LH increase and undesirable early oocyte ovulation detected during oocyte collection, which is one of the main causes of the high rates of cycle cancellations. Therefore, physicians frequently try to collect oocytes in an early period (between 30 and 32 hours), and this approach results in the collection of high numbers of immature oocytes due to early oocyte collection. 2 Kolibianakis et al. 20 evaluated the use of antagonists in the treatment of modified natural-cycle IVF treatment in patients with poor ovarian response. Recombinant FSH + GnRH antagonist were simultaneously administered when a follicle with a 14-mm was detected, and hCG was administered when the mean follicle diameter was ≥ 16mm. Oocytes could not be collected from 32.2% of the patients, and continuing pregnancy could not be achieved. 20 Although the oocyte collection time was 36 hours after hCG trigger in the case group, cycle cancellations due to premature ovulation was lower compared with the control group and compared with other studies, and the rates of the collected M _2_ oocytes was higher in the case group in our study.

Our study consists of patients with infertility problem related to diminished ovarian reserve who were observed with just one antral follicle at the beginning of the controlled ovarian stimulation. This group of patients cannot represent the whole infertile group with diminished ovarian reserve, but this can give some knowledge about this limited group of infertile patients with only one antral follicle. The low number of patients in both the case and control groups makes the other limitation of the present study. We could not find a difference in the pregnancy results in both groups, but this may be because of the low number of contributors. We assume that there will be better pregnancy results after finding more available oocytes for fertilization but cannot prove it with this limited number of patients. For clear evidence of better pregnancy results after addition of GnRH antagonist 24 hours after the ovulation triggering, there is need for more comprehensive studies with a large number of patients.

## Conclusion

The administration of a single-dose GnRH antagonist one day after hCG administration enabled a decrease in the rate of premature ovulation and an increase in the number of embryo transfers in patients with diminished ovarian reserve.
